# The CRZ1 transcription factor in plant fungi: regulation mechanism and impact on pathogenesis

**DOI:** 10.1007/s11033-024-09593-4

**Published:** 2024-05-10

**Authors:** A. Cacciotti, M. Beccaccioli, M. Reverberi

**Affiliations:** Department of Environmental Biology, Sapienza University of Rome, Rome, Italy

**Keywords:** Zinc finger, Responses to calcium variation, Environmental stresses, Mycotoxins, Fungal effectors

## Abstract

Calcium (Ca^2+^) is a universal signaling molecule that is tightly regulated, and a fleeting elevation in cytosolic concentration triggers a signal cascade within the cell, which is crucial for several processes such as growth, tolerance to stress conditions, and virulence in fungi. The link between calcium and calcium-dependent gene regulation in cells relies on the transcription factor Calcineurin-Responsive Zinc finger 1 (CRZ1). The direct regulation of approximately 300 genes in different stress pathways makes it a hot topic in host-pathogen interactions. Notably, CRZ1 can modulate several pathways and orchestrate cellular responses to different types of environmental insults such as osmotic stress, oxidative stress, and membrane disruptors. It is our belief that CRZ1 provides the means for tightly modulating and synchronizing several pathways allowing pathogenic fungi to install into the apoplast and eventually penetrate plant cells (i.e., ROS, antimicrobials, and quick pH variation). This review discusses the structure, function, regulation of CRZ1 in fungal physiology and its role in plant pathogen virulence.

## Introduction

Host-pathogen interaction is a key step in pathogenic development. Since the progression of the infection is based on different, discrete, steps, fine-tuning of various pathways is required especially in pathogen physiology as a response to host defense and the presence of a “master regulator” that precisely regulate the transcriptomic mechanism represent a focal point of the pathogen life. Because information is transmitted *via* signaling pathways that are not simple ON – OFF responses, modulation is achieved through a frequency modulator [1] based on oscillations in cytosolic calcium (Ca^2+^) concentrations, which act as ubiquitous signals and control a wide array of cellular functions [2]. Moreover, a transient increase in intracellular calcium levels is crucial for cell adaptation to external stress via the activation of the calmodulin (CaM)/ calcineurin (CaN) signaling pathway [3], which triggers (via dephosphorylation) the entry of the transcription factor Calcineurin-Responsive Zinc finger 1 (CRZ1) into the nucleus [4]. Here, the presence of a specific consensus sequence in the target gene promoter is defined as CDRE (calcineurin-dependent responsive element) through which the organism can mediate the transcriptomic response to adapt to external stimuli [5].

This review focuses on the structure of CRZ1, its role in the calcium cell survival (CCS) pathway, which is involved in a variety of life processes, and how it fits into the host-pathogen crosstalk. However, like the sorites paradox of when a heap of sand became only a grain, the singular events have already been revised in depth, but the role of CRZ1 in the inter-communication process is still not completely clear. Notably, pathogenic fungi must face a hostile and changing environment; from penetration to cell exploitation, the pathogen encounters several environmental insults, such as increased ROS concentration, alkalinization of the apoplast, and toxic phenolics *inter alia.* Adapting to this fast-changing environment requires tight control of gene expression and specifically of those genes involved in adaptation, such as antioxidation and detoxification, but concomitantly triggers the expression of genes related to virulence, which is a matter of simultaneous defense and attack. We believe that CRZ1 could play a leading or important role in orchestrating this strategy.

## Calcineurin responsive zinc finger CRZ1

The transcription factor CRZ1 belongs to the super-category of the zinc finger transcription factor (ZF-TF) and carries out its regulatory action through zinc finger motifs (ZF).Those domains are composed of one α-helix and two antiparallel β-strands, which also allows its categorization into different categories: the Cys2His2 family, that embraces hundreds of these proteins in eukaryotes, from yeast to humans [6], and the zinc cluster protein family (C6) that is only present in fungi with the unique CysX2CysX6CysX5-12CysX2CysX6-8Cys motif. In particular, the cysteine residues bind two zinc atoms to coordinate the correct folding of the DNA-binding domain [7], contributing to the correct protein function [8].

**Fig. 1:**
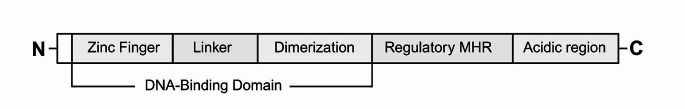
Domains of zinc cluster proteins: DBD, regulatory domain, and acidic region. The DBD consists of three subregions: zinc finger, linker, and dimerization domains. (Created with BioRender.com.)

CRZ1 belongs to the C6 family: a zinc finger with a DNA-binding domain (DBD) and six cysteine residues bound to two zinc atoms, categorized as a zinc cluster (ZFC), zinc binuclear cluster, or Zn(II)2Cys6 (Zn2C6) proteins. Those proteins have only one zinc finger domain with two zinc atoms, and shows the ability to bind DNA as monomers, homodimers, or heterodimers. ZFC proteins have been identified exclusively in fungi and are distributed at a ratio of 10:20:40 in chytrids-zygomycetes, basidiomycetes, and ascomycetes, respectively [9], although other ZFC proteins are also present [7]. The C6 family protein includes the transcription factor Gal4 of *Saccharomyces cerevisiae* [10], in which these six cysteine residues bind to two Zn(II) ions in a bimetal thiolate cluster [7]. Different functional domains have been identified in this protein, including a DNA-binding domain (DBD; residues 1–65), a dimerization domain (residues 65–94), three acidic activation domains, and a C-terminal region that interacts with the inhibitor Gal80p. The DBD consists of three regions: zinc finger, linker, and dimerization regions [7] (Fig. 1).

The site responsible for the Zn bond has two further “sub-structures” each formed by 3 Cys surrounded by basic amino acids and separated by a loop, which together form two short α-helices within which the two zinc atoms are positioned consequently bonded to 6 total Cys residues. This portion is normally found in the N terminal region [11]. The linker region, the most variable part of the protein, allows its extension on ssDNA, binding the phosphodiester backbone and generating an extremely rigid “scaffold” preventing the binding to off-target sequences [12]. In general, the dimerization region contains seven repeats, forming a conserved coiled-coil structure responsible for protein-protein interactions. The lack of this region, as in Ume6p of *S. cerevisiae*, indicates monomeric behavior [13]. Zn (II) 2Cys6 - DNA-binding domains typically interact with DNA-binding sites formed by conserved terminal trinucleotides. These trinucleotides feature a symmetrical arrangement and are separated by an internal sequence of variable lengths, ranging from 2 to 17 nucleotides. It is worth noting that these DNA-binding domains primarily interact with specific sections of the DNA [14] called, in CRZ1’s case, calcineurin dependent responsive elements or CDRE [15].

## CRZ1’s *passepartout*: the calcineurin dependent responsive element (CDRE)

### The consensus motif

The zinc finger domain of CRZ1 engages with the 24 bp CDREs sequence to initiate the expression of target genes [4]. In *S. cerevisiae*, the primary consensus site for CRZ1 binding is 5′-GNGGCKCA-3′ [16], whereas the proposed common DNA sequence bound by CRZ1 in *Trichoderma reesei* is 5′-GDGGCKBNB-3′ [17]. A recent analysis of promoters in *Candida albicans* revealed a distinct consensus motif [5′-GGAGGC(G/A)C(T/A)G-3′] that diverged from the putative CaCRZ1-binding motif [5′-G(C/T)GGT-3′] identified in a previous research [18]. In-depth studies have revealed that zinc cluster proteins recognize closely related elements, encompassing trinucleotide sequences presented either singly or in repeated patterns, whether symmetrical or asymmetrical. While CGG triplets are prevalent, variations within these binding elements have been documented, ensuring the capacity of each protein to perform unique regulatory functions [7]. As noted earlier, several factors exert influence over DNA targeting and binding of zinc cluster proteins. Within the protein structure, numerous components of the DNA-binding domain contribute significantly to interaction with the target DNA. Additionally, the nucleotides encompassing CGG triplets also exert a measure of influence on DNA-binding affinities [19]. However, two crucial factors that determine DNA-binding specificity are the orientation of CGG triplets and the distance between them.

### The target genes in fungi

CRZ1 governs a range of target genes that are responsible for maintaining ion equilibrium, sustaining cell wall integrity, driving lipid synthesis, managing protein degradation, and overseeing glucose metabolism. Its influence extends to species-specific genes, serving as a controller of both gene expression induction and inhibition. Additionally, CRZ1 is indispensable for orchestrating the response of the two P-type ATPases, PMC and PMR, to calcium ions to facilitate Ca^2+^ translocation from the cytoplasm to the vacuoles and Golgi apparatus. This finely tuned transport safeguards the intracellular calcium balance [20].

In yeast *pmc* and *pmr* gene expression displays an increased in response to Ca^2+^ stimuli. However, CRZ1 mutants exhibit reduced activation of these genes [16, 21]. This attenuation leads to disrupted calcium equilibrium, potentially explaining the calcium sensitivity observed in CRZ1 mutants. CRZ1 dependence extends to other genes such as *ena1*, *ena2*, and *ena3*, coding for plasma membrane Na^+^/Li^+^-ATPases that ensure yeast viability at elevated concentrations of these ions. Their induction depends on CaN and CRZ1 interactions [22]. The CaN-CRZ1 pathway also regulates ion homeostasis-related genes such as *mep1*, *enb1*, *pho84*, *pho89*, and *kha1* [16]. In encounters with external stressors, cell wall integrity relies on β-1,3 glucan synthase (FKS) and chitin synthase (CHS). Disruption of CRZ1 prompts irregular expression of *fks* and *chs* [23].

In plant pathogens, as *Aspergillus flavus*, the CaN-CRZ1 axis extends its influence on other cell wall maintenance genes such as *crh1*, *rho1*, *scw10*, and *kre6* [14]. Notably, CRZ1’s role in *Penicillium oxalicum* encompasses cellulase synthesis regulation through genes such as *cbh1*, *eg1*, and *eg2* [24].

Furthermore in *A. flavus*, lipid and sterol metabolism-associated genes (*sur1*, *csg2*, *ysr3*, *erg26*, *hes1*, *and plb3*), along with vesicular transport-related genes (*gyp7*, *ypt53*, *yip3*, *pep12*, *rvs161*, *she4*, *cvt17*, *cvt19*, *and vps36*), are also CRZ1-regulated. Collectively, these genes enable proficient membrane function and efficient substance delivery to the cell surface [14]. These evidences in plant pathogenic fungi show the role of calcium homeostasis as pivotal for orchestrating effective responses to diverse stimuli.

## IN-N-OUT: mechanism of CRZ1 activation

**Fig. 2:**
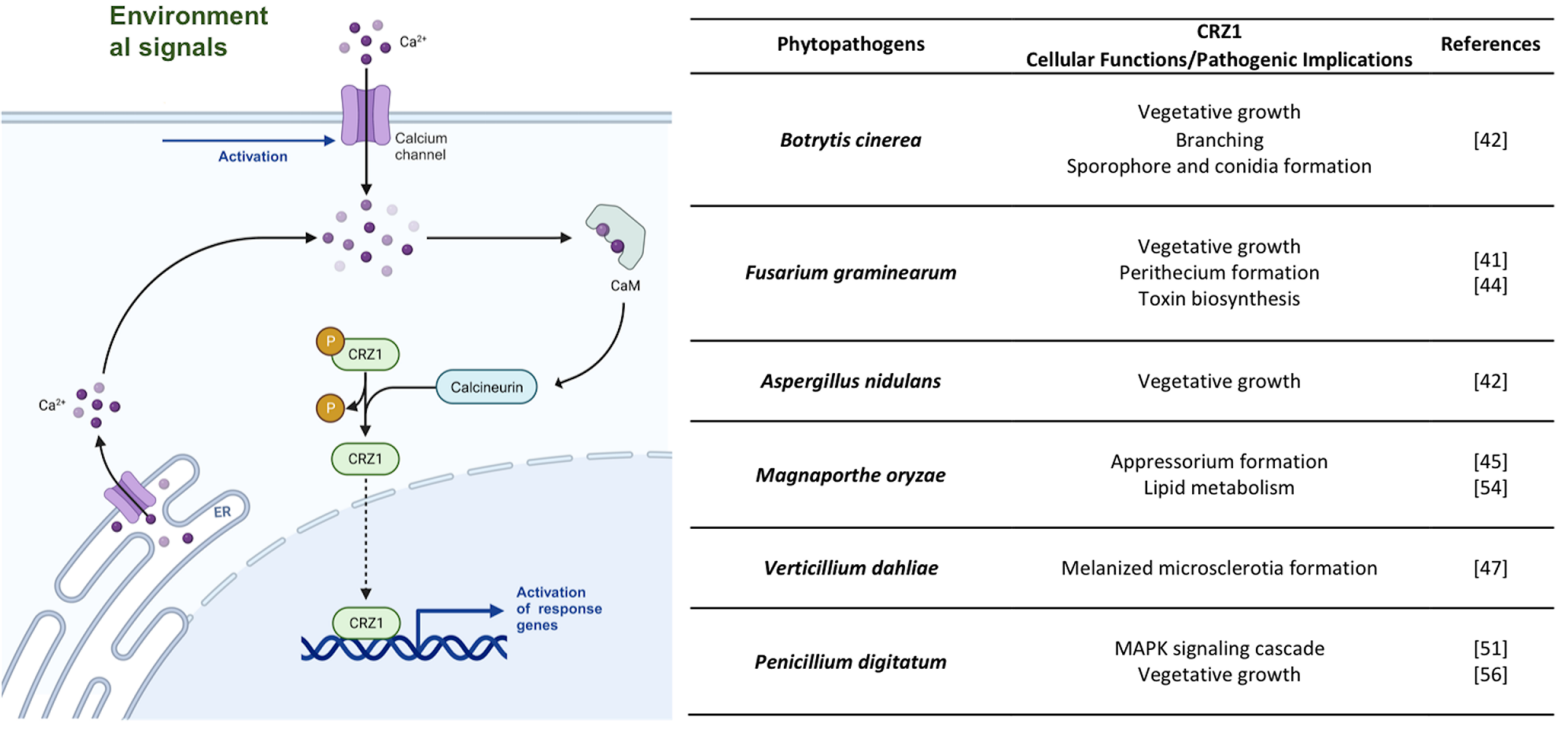
**a**, regulation of CRZ1 in the presence of environmental stressors (left panel). The perception of signals leads to the accumulation of Ca^2+^ in the cytoplasm through the opening of Ca^2+^ channels (e.g., in the endoplasmic reticulum or vacuole). This process activates calmodulin (CaM), which phosphorylates calcineurin, which in turn, dephosphorylates CRZ1 that shuffles into the nucleus to activate the transcription of target genes (Created with BioRender.com.), **b**, list of cellular functions and pathogenesis implication of CRZ1 in plant pathogenic fungi

Calcium signaling plays a pivotal regulatory role throughout fungal growth and development. Defects in this pathway trigger anomalies in different facets of fungal cells, including reproductive development, polar growth, cell differentiation and division, stress responses, and programmed cell death [25]. Consequently, preserving the intracellular calcium balance is a crucial factor in determining cell survival. Under normal physiological conditions, the fungal cell cytoplasm harbors a modest concentration of Ca^2+^ ranging from 50 to 100 nM [25]. Eukaryotes manage cellular calcium stability through an intricate regulatory system that involves diverse Ca^2+^ channel proteins, pumps, transporters, and relevant enzymes [26]. Positioned predominantly on the plasma membrane or within various subcellular organelles, these constituents collaboratively manage Ca^2+^ intake from extracellular and intracellular reservoirs, thereby orchestrating the equilibrium between cytoplasmic and organelle-specific Ca^2+^ levels [27]. The calcium/calcineurin pathway, a well-conserved element spanning from mammals to yeasts [28], involves calcineurin, a phosphatase that triggers stress responses and preserves drug resistance. This function is enacted through protein-protein interactions or genetic interplays involving CRZ1, which governs the activation of over 100 genes [29]. Although calcium bursts are recurring observations in various cell types, such as yeast [30], the relationship between cytosolic calcium concentration and CRZ1 localization within individual cells is yet to be scrutinized. Mechanistic models of CRZ1 regulation via calcium signaling do not anticipate the oscillation of cytoplasmic calcium concentration ([Ca^2+^]_cyt_) induced by variations in the external calcium concentration ([Ca^2+^]_ext_) [31]. Additionally, although the average nuclear localization of CRZ1 increases with elevated [Ca^2+^]_ext_ [32], [Ca^2+^]_cyt_ is rigorously regulated and maintains similarity across a broad range of [Ca^2+^]_ext_ [33]. This discrepancy implies that the frequency of CRZ1 pulsatility, which heightens with [Ca^2+^]_ext_ rising [32], is unlikely to mirror the variance in the average [Ca^2+^]_cyt_. *Hsu et al.* [1] posited that the connection between [Ca^2+^]_cyt_ and CRZ1 pulsations resembles that of a noisy analog-to-digital converter: heightened calcium bursts correspond to increased CRZ1 pulses. Prior investigations of CRZ1 pulsatility suggest that CRZ1 pulses are actively generated, rather than passively tracking fluctuations in [Ca^2+^]_cyt_ [32]. Instead, individual CRZ1 molecules have been proposed to gauge [Ca^2+^]_cyt_ with time delay. This interpretation potentially elucidates the observed correlation between a high affinity calcineurin docking site on CRZ1 and an increased pulsing frequency [32], where greater affinity facilitates longer oscillations following a calcium burst. A proteome-wide screen was conducted using movies to systematically identify localization-based pulsing behaviors in *Saccharomyces cerevisiae* [34], analyzing all genes in a previously developed library of 4,159 strains containing fluorescent protein fusions [22]. Interestingly, CRZ1 is the only found nonredundant or paralogous pulsed transcription factor. The prevalence of pulsing in various biological systems across different species implies that pulsing could be a widespread solution to many biological challenges. Indeed, it has been shown to proportionally regulate entire regulons of target genes [32] and providing a temporal mode of regulation that facilitates this and other functions [35]. Calcineurin interacts with the transcription factor through the calcineurin docking domain PIISQ [36] and a serine-rich region housing multiple serine residues. This region serves as a target for CRZ1 dephosphorylation, influencing its localization and phosphorylation levels [37]. In the absence of external stress or other stimuli, CRZ1 resides in the cytoplasm. However, elevated Ca^2+^ concentrations trigger CaN activation, leading to CRZ1 dephosphorylation and its subsequent migration to the nucleus for the regulation of target genes (Fig. 2a).

CRZ1 shuffling dynamics arise from the presence of both a nuclear localization signal (NLS) and nuclear export signal (NES). In quiescent cells, CRZ1 predominantly occupies the cytosol, because the prevailing rate of nuclear export exceeds that of nuclear import. Upon the activation of calcineurin-dependent signaling, CRZ1 swiftly relocates to the nucleus, thereby initiating gene expression. The dephosphorylation of CRZ1p induces a conformational alteration that unveils its NLS, allowing it to interact with importin NMD5p [38]. This dephosphorylation also inhibits the interaction between CRZ1’s NES and the export receptor MSN5p, thereby curbing nuclear export. Calcineurin-mediated dephosphorylation of CRZ1 serves a dual purpose: to increase the nuclear import rate and diminish the nuclear export rate. These combined effects expedite CRZ1’s nuclear accumulation upon calcineurin activation, with continuous calcineurin-mediated dephosphorylation “vital” for maintaining CRZ1’s nuclear localization. The termination of signaling prompts CRZ1’s rapid cytosolic return, driven by its re-phosphorylation [37]. However, in species such as *C. glabrata* and *C. neoformans*, CRZ1 activation occurs through a calcineurin-independent route [4]. In one instance, a microarray analysis identified 33 genes under CRZ1 regulation via a calcineurin-independent mechanism. Of these, 16 were upregulated and 17 were downregulated upon exposure to the calcineurin-inhibiting drug FK506 [39]. In *C. dubliniensis*, CRZ1-directed control of thigmotropism is calcineurin-independent [40]. *S. cerevisiae* shows a distinct angle, where the kinase HRR25, with roles encompassing the DNA damage response, mitosis, and vacuole transport, engages with CRZ1, phosphorylating it to elicit alterations in its localization [41]. The ability to modulate diverse stress response pathways positions CRZ1 as a focal point in plant pathology research, given its multifaceted regulatory involvement in governing virulence within host-pathogen interactions.

## CRZ1-mediated response to host stressors

The stressors inherent in host-pathogen interactions encompass a spectrum of variations, including oxidative stress, pH fluctuations, and interference agents targeting the cell wall. In response, fungi have evolved diverse strategies to swiftly perceive these signals and mitigate the resulting damage. The transcription factor CRZ1 has emerged as a pivotal player, activated by the surge in Ca^2+^ levels induced by stress, orchestrating the regulation of pertinent gene expression. Indeed, CRZ1 mutants exhibit heightened sensitivity to ion stress owing to the translocation of dephosphorylated CRZ1 into the nucleus, triggering the expression of multiple genes involved in calcium ion stress responses, such as *pmc* and *pmr* [16]. However, susceptibility to cation ion stressors, like Na^+^, Li^+^, Mg^2+^, and Mn^2+^, varies across fungal species with CRZ1 deletions. For instance, in *Aspergillus fumigatus*, the absence of CRZ1 leads to high sensitivity to Mn^2+^ but diminished sensitivity to Na^+^ and Li^+^. In contrast, in *Magnaporthe grisea* the knockout of *∆Crz1* was insensitive to Na^+^, Li^+^, and Mn^2+^ [55]. Conversely, in *Botrytis cinerea* the absence of *Crz1* gene generates mutant with a pronounced sensitivity to these four ion stresses; however, the addition of Mg^2+^ restored growth impairments and cell wall integrity [42]. These insights underscore the commonality between CRZ1-regulated ion stress responses and ion homeostasis in fungi, albeit with species-specific nuances. Evidently, CRZ1’s sensitivity to oxidative stress transcends species barriers, as has been confirmed in various organisms including *B. cinerea* [42], *Metarhizium acridum* [56], and *Penicillium digitatum* [56] (Fig. 2). Furthermore, CRZ1’s role in yeast tolerance to elevated pH levels is well established. Alkaline conditions elicit the expression of alkaline pH-responsive genes, such as *ena1*, *pho84*, *pho89*, and *pho12* [58]. Under extreme pH levels (pH 3 or 9), Δ*BcCrz1* exhibits diminished colony growth rate, akin to ion stress scenarios, and exogenous Mg^2+^ supplementation restores growth at pH 9, although the growth defect remains unresolved at pH 3 [42]. Another facet of the Δ*Crz1* mutant is its heightened sensitivity to cell wall inhibitors. Notably, the growth of CRZ1 mutants in *P. digitatum*, *Magnaporthe oryzae*, and *B. cinerea* is markedly impaired in media containing SDS, CR, or CFW [42, 54, 57]. Interestingly, in *Valsa pyri,* *∆VpCrz1* exhibited significantly increased mycelial growth on CM agar medium containing SDS, CR, or CFW compared with the wild-type strain, diverging from previous reports [47]. Correspondingly, the CRZ1 mutant showed SDS resistance in the human pathogenic fungus *Candida lusitaniae*, suggesting CRZ1’s negative regulation of cell membrane integrity. Intriguingly, its response to SDS operates through an unknown mechanism, independent of CaN [59].

## The bad, the ugly and the CRaZy: involvement in pathogenesis of plant fungi

As previously elucidated, the CSS signaling cascade maintains sway over fungal growth, development, and pathogenicity. The absence of CRZ1 causes aberrations in the vegetative growth of most plant pathogenic fungi (Fig. 2b). A prime example is the *∆BcCrz1* mutant of *Botrytis cinerea*, which exhibits compromised mycelial growth and unusual branching in CM medium [42]. In addition, *Fusarium graminearum* and *Aspergillus nidulans* hinder vegetative growth following CRZ1 loss [41, 42]. Crucial to fungal life, conidia formation and development are intimately linked to CRZ1. For instance, *∆BcCrz1* fails to generate sporophores or conidia [42, 43], whereas *∆FgCrz1* presents impaired perithecium formation, thereby affecting sexual development [44]. A noteworthy instance is observed in *A. nidulans*, where a pressure-sensing mechanism on the cell wall triggers calcium channel opening, activating the CNA/CRZ1 complex and propelling mycelial polar growth. Beyond this, CRZ1’s role extends widely, encompassing mycelial growth, morphological transformation, spore and appressorium formation, and serving as a precursor for pathogenicity [4]. In *Magnaporthe oryzae*, CRZ1 deleted strains exhibit reduced pathogenicity, which is mainly attributed to decreased appressorium swelling pressure, resulting in osmotic damage owing to disrupted lipid metabolism [45]. *Verticillium dahliae*, implicated in Verticillium wilt disease in smoke trees [46], generates melanized microsclerotia and in the Δ*VdCrz1-2* mutant, microsclerotia formation was markedly compromised, with accumulated melanin and increased fragility [47]. Furthermore, expression of the hydrophobin gene *vdh1* is pivotal for early microsclerotia development [48] and, with other melanin biosynthesis genes, is significantly downregulated in Δ*VdCrz1-2.* The addition of Mg^2+^ to low-Mg^2+^ basal medium restores the abnormal microsclerotia phenotype, underscoring the pivotal role of the calcineurin-CRZ1 signaling machinery in host plant infection [47]. *P. digitatum*, a citrus fruit pathogen, exhibits diminished virulence and a ~ 35% reduction in lesion size due to the *∆PdCrz1* mutation [49]. *V. pyri*, which inflicts canker disease on apples and pears [50], witnesses reduced diameter of wounded pear fruits and 1-year-old branches in the Δ*VpCrz1* mutant. The attenuated virulence is attributed to the CRZ1/MAPK signaling cascade [51]. In *(B) cinerea*, the Δ*BcCrz1* mutant experiences compromised cell wall integrity, leading to defective mycelium-derived infection and reduced lesion diameter in infected bean plant leaves. However, as reported for the *V. dahliae* CRZ1 mutant, Mg^2+^ supplementation enhanced cell wall stability in Δ*BcCrz1* hyphae and mitigated penetration defects [42]. *F. graminearum*, the causative agent of Fusarium head blight (FHB) in wheat and cereals, generates mycotoxins such as deoxynivalenol (DON) and zearalenone (ZEA) in infected grains [51, 52]. The *ΔFgCrz1A* mutant showcases attenuated virulence, struggles to propagate in a neighboring spikelet, and displays reduced expression of the DON biosynthesis gene *tri*, resulting in decreased DON production [44]. In *M. oryzae*, lipid droplet transportation from conidia to nascent appressorium is disrupted in Δ*MoCrz1*, leading to a decreased appressorial penetration rate and early lipid droplet degradation upon infection [53, 54]. While CRZ1 indeed assumes a conserved role in fungal virulence or pathogenicity, distinct mechanisms govern these effects, as revealed through studies on fungal pathogen infection mechanisms.

## Relation between CRZ1 and mycotoxin biosynthesis

Mycotoxins are harmful humans/animals’ secondary metabolites produced by filamentous fungi. Defining their ecological role is challenging. Only in a few cases, among which aflatoxins and deoxynivalenol, their role in fungal lifestyle has been suggested: aflatoxins should provide stationary-phase *A. flavus* colonies with an extended lifespan owing to their high antioxidant abilities [60], while deoxynivalenol appears to be crucial for rachis penetration during *F. graminearum* colonization of ovary tissues into wheat spikelets [61]. Interestingly, the synthesis of both mycotoxins is apparently controlled by CRZ1. In *F. graminearum*, the lack of CRZ1 (in the *ΔFg01341* mutant) causes dramatic defects in the ability to colonize flowering wheat heads and corn silks, which is consistent with the reduction in deoxynivalenol production demonstrated in this strain [44] (Fig. 2b). In *A. flavus*, deletion of the CRZ1-orthologue *crzA* causes a severe impairment in the ability to colonize maize kernels and aflatoxins production [62]. These few examples suggest us that CRZ1 by exerting a role also in mycotoxin synthesis could effectively represent a “hub” for orchestrating general responses to stresses: specifically, the host poses various threats to fungal survival, forcing responses that requires the correlation among diverse pathways int pathogenic fungi some of which (e.g., mycotoxins) are not forcedly or closely related to virulence.

## Conclusions

As a vital transcription factor in the calcium signaling pathway, CRZ1 is extensively preserved in fungi and performs a crucial function in growth, development, stress tolerance, and pathogenicity. The link between stress response and transcriptional regulation makes CRZ1 a key element in the survival mechanism of the pathogen, particularly in adverse conditions, such as the interface with the host. CRZ1 can regulate different responses, particularly which gene cluster; however, some pieces are missing. The presence of a small binding motif, where only three base pairs seem to be fundamental, makes analysis very difficult, and an *in silico* approach can be misleading and limited in identifying subtle changes in complex binding motifs under different conditions. To overcome this limitation, it is crucial to use tools that enable sensitive identification with a single base-pair resolution of complex alterations to core-binding motifs. Another limitation is the nature of the zinc finger, which can regulate different genes in the presence of a co-factor. This different regulation mechanism increases the analysis difficulty since other types of transcription factor can bind to different sequences making the “predictive” approach useless. Vital information can be obtained using an immune precipitation approach to identify other proteins with which a protein can collaborate. Furthermore, given the variety of pathways that CRZ1 regulates, it is necessary to conduct a multi-omics analysis combined with transcriptome sequencing to understand the full metabolic pathway in fungi.

## Data Availability

No datasets were generated or analysed during the current study.
